# Maternal smoking and preterm birth: An unresolved health challenge

**DOI:** 10.1371/journal.pmed.1003386

**Published:** 2020-09-14

**Authors:** Sarah J. Stock, Linda Bauld

**Affiliations:** Usher Institute, University of Edinburgh, Edinburgh, United Kingdom

Maternal exposure to tobacco smoke in pregnancy is a key modifiable risk factor for baby death and disability. Smoking is linked to preterm birth (birth before 37 weeks’ gestation), stillbirth, and neonatal mortality, as well as to miscarriage, fetal growth restriction, and infant morbidity [1]. The worldwide prevalence of maternal smoking in pregnancy is 2%, with Europe having the highest prevalence at 8% [2]. Although rates of maternal smoking in pregnancy are decreasing in many high-income countries [2], this decline is slower among women of lower socioeconomic status, contributing to health inequalities [3]. In certain low- and middle-income countries, maternal smoking rates are static or rising [4–6].

In this issue of *PLOS Medicine*, two studies [7,8] provide new insights into the implications of exposure to tobacco smoke in pregnancy for perinatal and childhood outcomes. Buyun Liu and colleagues studied preterm birth in relation to timing and intensity of maternal smoking in more than 25 million singleton mother–infant pairs using United States birth certificate data [7]. The size of this “mega-cohort” allowed exploration of whether incremental increases of 1–2 cigarettes per day were associated with increases in preterm birth. Compared to nonsmokers, any maternal smoking during the three months prior to conception and continued into the first trimester of pregnancy was associated with increased preterm birth (odds ratio [OR] 1.17 [95% CI 1.16–1.19]). This risk increased if maternal smoking continued during the second trimester (OR 1.45 [1.45–1.46]). Women who quit smoking during pregnancy still had an increased risk of preterm birth, even if levels of smoking were low and they stopped early in pregnancy. For example, compared to nonsmokers, women who smoked 1–2 cigarettes a day and quit in the first trimester had an increased risk of preterm birth (OR 1.13 [1.10–1.16]). In contrast, if they quit smoking in the three months before pregnancy, even heavy smokers of 20 or more cigarettes per day had a similar risk of preterm birth to that of nonsmokers (OR 1.01 [0.99–1.03]). The authors conclude that there is no safe level for cigarette smoking in pregnancy.

Elise Philips and colleagues found a different pattern of smoking and preterm birth in an individual participant data meta-analysis of 220,000 births from 28 cohort studies, in which smoking status was determined from questionnaires [8]. Compared to nonsmokers, mothers who smoked in the third trimester of pregnancy were at increased risk of preterm birth. However, the effect size was lower than in Liu’s study [7], with an OR of 1.08 (1.02–1.15). In contrast to Liu’s findings [7], smoking confined to the first trimester of pregnancy was not associated with preterm birth when compared to nonsmokers (OR 1.03 [0.85–1.25]). Furthermore, no dose response was seen with increasing or decreasing cigarette intake between first and third trimesters.

Philips and colleagues additionally explored the relationship between smoking and being small for gestational age (SGA) at birth and overweight in childhood [8]. Whereas maternal first trimester smoking was associated with childhood overweight (OR 1.17 [1.02–1.35]) but not SGA (OR 0.99 [0.85–1.15]), smoking in later pregnancy was associated with both childhood overweight (OR 1.42 [1.35–1.48]) and SGA (OR 2.15 [2.07–2.23]). Reducing the number of cigarettes from first to third trimester lowered the risks of delivering SGA infants, but risks were still higher compared with nonsmoking mothers. Mothers who increased the number of cigarettes from first to third trimester had increased risk of an SGA infant compared with those who did not.

Several factors may explain the different patterns of association between smoking and preterm birth seen in the two studies. First, at 4.7%, the population risk of preterm birth in the Philips study, in which most of the cohorts were European [8], was less than half that of Liu’s US-based study (9.3%) [7,8]. Second, the sample size for analyses of cessation, increasing, or decreasing cigarettes smoked between first and third trimester was much smaller in Philips’ study [8] and, at only 1% of the entire cohort (around 2,200 women with 120 preterm births), may not be representative at population level. The low numbers resulted from only around half of the included cohorts having data on both early and late pregnancy cigarette consumption. Third, in the Philips study, smokers who quit prepregnancy were included as nonsmokers, whereas in the Liu study, prepregnancy smokers were considered separately. Finally, cohorts in the Philips meta-analysis collected late pregnancy smoking data in the third trimester [8]. This can be problematic, as most preterm births occur in the third trimester. Liu and colleagues restricted analysis to second-trimester smoking to avoid this [7,8].

Despite their differences, both studies [7,8] add compelling evidence to the idea that there is a dose–response relationship between smoking in pregnancy and preterm birth. The more and the longer women smoke in pregnancy, the higher the associated morbidity. There will also be higher numbers of babies who die, as preterm birth is the major cause of neonatal mortality, and SGA is strongly associated with stillbirth. This message needs to be clearly conveyed to pregnant women and health professionals so that the relevance of surrogate health outcomes is not misinterpreted. Having a “small baby” may not be seen as a bad thing or even, erroneously, be considered advantageous for birth. Health messages should also be directed to wider audiences than just pregnant women and those that care for them. As beliefs about smoking are strongly influenced by family, friends, and peers, risk messages from social networks are frequently more effective than those delivered by health professionals [9].

Pregnancy is a time when interventions for smoking cessation might be most effective. It is purported that women are more likely to quit smoking in pregnancy than at any other period in their lives [10]. There are certainly opportunities for improvement, with three-quarters of prepregnancy smokers continuing to smoke in early pregnancy and 85% of those that smoke in early pregnancy continuing into late pregnancy [7,8]. Behavioral support for smoking cessation is recommended as part of antenatal care in many countries and endorsed by guidance from WHO [11]. This should be delivered by staff who have received appropriate training but delivered in a flexible way, tailored to the needs of pregnant women. Some countries combine behavioral support with nicotine replacement therapy, which has been shown to be effective in the general adult population. However, single-product nicotine replacement therapy has not been shown to be effective during pregnancy [12], and research is now ongoing to explore this further [13].

Evidence from ongoing trials of promising adjuvant approaches, such as electronic cigarettes [14] and financial incentives [15], may be key to improving quit rates but will require political will to implement if effective. There are, however, enormous potential benefits from reducing smoking in pregnancy, both in terms of women’s and children’s health and in savings to health services. In the United Kingdom alone, maternal and infant healthcare costs attributed to smoking are estimated at £20–£87.5 million per annum [16]. A concerted effort across multiple sectors is required to prevent this harm and protect the health of future generations.

## Abbreviations

OR: odds ratio
SGA: small for gestational age
